# Late-Onset Meningeal Metastases in Liposarcoma: A Case Report and Review of the Literature

**DOI:** 10.1155/SRCM/2006/23039

**Published:** 2006-12-24

**Authors:** Michelle Ferguson, William Stewart, Fiona Cowie, Jeff White

**Affiliations:** ^1^Department of Oncology, Ninewells Hospital and Medical School, Ward 32, Dundee DD1 9SY, UK; ^2^Department of Neuropathology, Southern General Hospital, 1345 Govan Road, Glasgow G51 4TF, Scotland, UK; ^3^Beatson Oncology Centre, Dumbarton Road, North Glasgow University Hospitals Division, Glasgow G11 6NT, Scotland, UK

## Abstract

Intracranial metastases from liposarcoma are rare and almost always preceded by the development of systemic tumour spread. We report here a case of liposarcoma with spread to the cranial nervous system 23 years after treatment of the primary tumour. The literature on brain metastases from soft tissue sarcoma is also reviewed.

## CASE REPORT

A 48-year old Caucasian female with active rheumatoid arthritis was diagnosed with a low-grade myxoid liposarcoma of the left thigh in 1981. This was treated with surgery followed by radiotherapy. In 1991, she was discharged from routine oncological followup. In 1998, she was rereferred by her GP for investigation of a left groin mass. Biopsy of the groin mass confirmed recurrent liposarcoma and restaging investigations confirmed that the lesion was isolated. Surgery and radiotherapy (5000 Gy in 25 fractions) were used to treat the recurrent liposarcoma. The pathology report revealed that the tumour had transformed into a “high-grade myxoid/round-cell liposarcoma surrounding the iliac artery and vein, involving the deep resection margins.”

Over the next 6 years, the patient continued on close outpatient followup with regular chest X rays. During this time, persistent left leg oedema became problematic. In addition, her rheumatoid arthritis remained symptomatic despite treatment with hydroxychloroquine, methotrexate, and indomethacin.

**Figure 1:**
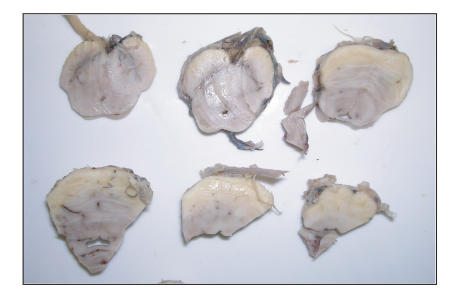
Gross postmortem image of mid brain showing thickened meninges.

**Figure 2:**
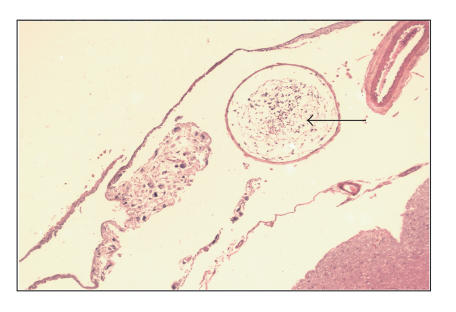
Liposarcoma cells seen within a blood vessel in the subarachnoid space.

**Figure 3:**
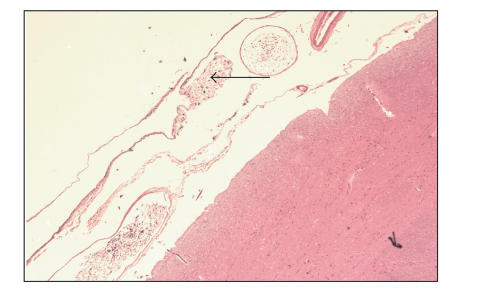
Liposarcoma in subarachnoid space.

In October 2004, the patient was admitted as a medical emergency with a 5-day history of nausea and vomiting, severe occipital headache, photophobia, and diplopia. In addition, she described a strange sensation in her tongue with impaired tongue movement. On examination, a 3 cm × 3 cm smooth mobile cervical neck node was noted in addition to a left VI cranial nerve palsy, a right XII cranial nerve pals, y and hypotonia of the left side of the tongue. A lumbar puncture, CT brain, and blood tests including a vasculitic screen and viral serology were normal. An MRI demonstrated a “high signal at apex of the temporal bone” but was otherwise normal. Over the next 10 days, the patient continued to complain of a persistent dull throbbing headache and developed a right-sided VI nerve palsy. FNA of the neck node confirmed metastatic liposarcoma and a CT scan of the chest/abdomen and pelvis revealed “widespread tumour deposits in the right psoas muscle and pelvis.” The lungs were clear of metastatic disease. A repeat MRI done 3 weeks following her admission this time showed that “at the skull base, the clivus gives an abnormal marrow signal with infiltration, most likely due to metastatic disease.” Due to intractable symptoms, she went on to receive palliative radiotherapy to the base of skull (30 Gy in 10 fractions). A repeat CT scan done 6 weeks after the last scan showed progressive disease in the abdomen and pelvis with new lung metastases. Whilst waiting to commence palliative chemotherapy, the patient developed neurogenic dysphagia and died from aspiration pneumonia. At postmortem, meningeal thickening was noted in the area of the mid brain (Figure 1). Further neuropathological examination revealed liposarcoma cells within blood vessels in the subarachnoid space (Figure 2) in addition to deposits of metastatic liposarcoma in the subarachnoid space itself (Figure 3).

## DISCUSSION

Brain metastases from soft tissue sarcoma (STS) are rare, occurring in 1–3% patients [1–3]. Autopsy series have demonstrated a higher rate of occult intracerebral metastases ranging from 3% to 16% [1, 2, 4]. When diagnosed in the symptomatic patient, brain metastases are usually preceded by pulmonary metastases [3].

Previous studies have identified leimyosarcoma, liposarcoma, and malignant fibrous histiocytoma (MFH) as the subtypes of STS most likely to metastasize to the brain [3]. Wronski et al in 1995 identified embryonal rhabdomyosarcoma as the STS subtype most likely to spread to the brain in his review of patients ranging in age from 2.6 to 68 years [5]. Embryonal rhabdomyosarcoma is the commonest STS amongst children and young adults and this may explain the difference between these reports.

In addition to the tumour subtype in STS, the site of initial disease also appears influential in determining the most likely sites of distant metastases. The first site of distant metastasis for patients with extremity and trunk STS is usually the lung with local recurrence rarely a problem in these patients [6, 7]. In contrast, patients with retroperitoneal and visceral STS, of the same histological type, tent to recur locally rather than at distant sites. These patients die of local failure rather than surviving long enough to succumb to metastatic disease [8, 9]. Early surgical intervention of isolated intracranial metastases prolongs disease-free and overall survival [3, 10] and remains the standard treatment. External beam radiotherapy offers local control in these patients but does not translate into increased survival [10]. Chemotherapy for the treatment of STSBM (soft tissue sarcoma brain metastases) has been disappointing with the exception of an isolated report of complete remission of brain metastases in one patient treated with Doxorubicin and Dacarbazine [11]. Unfortunately, the other sites of visceral disease in this patient failed to respond to this regimen.

This case describes the development of intracranial metastases in a patient treated 23 years previously for a liposarcoma of the lower limb. Postmortem findings confirmed that this patient's neurological signs and symptoms were due to meningeal metastatic disease of the same histological subtype as the original tumour. The brain metastases became clinically apparent 23 years after treatment of the initial primary. Such a long interval between treatment of the primary tumour and CNS relapse has been described elsewhere in patients with prolonged survival, either in association with the STS having a long natural history or as a result of extended disease-free survival from effective local treatment of the primary tumour [12, 13]. Others have described an increase in CNS metastases in the setting of prolonged remission induced by chemotherapy [1, 12]. This patient did not receive chemotherapy as part of her treatment as her brain metastases were only diagnosed at postmortem and uncharacteristically were not preceded by the development of pulmonary metastases.

This case illustrates that STS can relapse in the CNS without the presence of widespread disseminated disease. CNS relapse can occur many years after treatment of the primary tumour and a high index of suspicion should be maintained in any patient with an STS presenting with neurological signs and symptoms. Early diagnosis and appropriate treatment of selected patients with STSBM can improve survival.

## References

[1] Bryant BM, Wiltshaw E (1980). Central nervous system involvement in sarcoma. A presentation of 12 cases, a review of the literature, and a discussion of possible changing patterns with the use of chemotherapy, placing special emphasis on embryonal tumours. *European Journal of Cancer and Clinical Oncology*.

[2] Lewis AJ (1988). Sarcoma metastatic to the brain. *Cancer*.

[3] Espat NJ, Bilsky M, Lewis JJ, Leung D, Brennan MF (2002). Soft tissue sarcoma brain metastases: prevalence in a cohort of 3829 patients. *Cancer*.

[4] Vezeridis MP, Moore R, Karakousis CP (1983). Metastatic patterns in soft-tissue sarcomas. *Archives of Surgery*.

[5] Wroński M, Arbit E, Burt M, Perino G, Galicich JH, Brennan MF (1995). Resection of brain metastases from sarcoma. *Annals of Surgical Oncology*.

[6] Lewis JJ, Brennan MF, Sabiston D (1997). Soft tissue sarcomas. *The Biological Basis of Modern Surgical Practice*.

[7] Gadd MA, Casper ES, Woodruff JM, McCormack PM, Brennan MF (1993). Development and treatment of pulmonary metastases in adult patients with extremity soft tissue sarcoma. *Annals of Surgery*.

[8] Brennan MF (1997). The enigma of local recurrence. The Society of Surgical Oncology. *Annals of Surgical Oncology*.

[9] Lewis JJ, Leung D, Woodruff JM, Brennan MF (1998). Retroperitoneal soft-tissue sarcoma: analysis of 500 patients treated and followed at a single institution. *Annals of Surgery*.

[10] Singer S, Corson JM, Gonin R, Labow B, Eberlein TJ (1994). Prognostic factors predictive of survival and local recurrence for extremity soft tissue sarcoma. *Annals of Surgery*.

[11] Haft H, Wang GC (1988). Metastatic liposarcoma of the brain with response to chemotherapy: case report. *Neurosurgery*.

[12] Espana P, Chang P, Wiernik PH (1980). Increased incidence of brain metastases in sarcoma patients. *Cancer*.

[13] Arepally G, Kenyon LC, Lavi E (1996). Late onset of isolated central nervous system metastasis of liposarcoma - a case report. *American Journal of Clinical Oncology*.

